# Policy and Food Consumption: What Nutrition Guidelines Are Swiss Children Meeting and What Determines Adherence?

**DOI:** 10.3389/fnut.2021.641799

**Published:** 2021-06-04

**Authors:** Natalie Rangelov, Raquel Nogueira Avelar e Silva, L. Suzanne Suggs

**Affiliations:** ^1^BeCHANGE Research Group, Institute of Public Health and Institute of Public Communication, Universitá della Svizzera italiana, Lugano, Switzerland; ^2^Department of Clinical Medicine-Department of Clinical Epidemiology, Aarhus University Hospital, Aarhus, Denmark; ^3^Swiss School of Public Health, Zurich, Switzerland

**Keywords:** children, eating behavior, dietary guidelines, guideline adherence, determinants

## Abstract

**Objectives:** To describe the adherence of the children to the Swiss Society for Nutrition (SSN) dietary guidelines, assess determinants of adherence, and compare these findings with a previous study in the same population.

**Methods:** Data from 312 children ages 5–12 were collected through a survey and a 2-day food record. The associations of children- and parent-related factors with adherence of the children to guidelines were assessed by logistic regression analyses.

**Results:** SSN guidelines were not met for any food category, although there were improvements: vegetables (4.5% in this study vs. 0% in the previous study), sweets, snacks, and soft drinks (SSD) (12.5 vs. 9.5%), and fruit (45.5 vs. 10.4%). Higher Body Mass Index (BMI) in children was associated with higher adherence to guidelines for protein intake. Higher parental BMI was associated with higher adherence to vegetables. Parental lower educational level was associated with higher adherence for cereal.

**Conclusion:** Despite improvements since the last eating behavior assessment in this population, children consume too little fruit, vegetables, cereal, and milk and dairy products, and too much SSD and proteins. Further efforts are needed to promote healthy eating to children and achieve adherence to guidelines.

## Introduction

Poor nutrition is one of the determinants of overweight and obesity, which are associated with an increased risk for type 2 diabetes, high cholesterol, hypertension, cardiovascular diseases, back pain, and asthma among others (1, 2). Studies have also shown that childhood obesity is associated with poorer cognitive skills and lower school achievements, as well as lower self-esteem and other mental health issues (3–9).

Children- and parent-related factors, such as gender, level of education, and body mass index (BMI), have been shown to be associated with the eating behavior of the children (10–13). A study showed that the highest daily intake of fruit was reported in girls in Albania (55%) and in Denmark and Switzerland (both at 51%). For vegetables, data were similar: girls consumed more vegetables than boys. Regarding the level of education, higher educated adults consumed more fruit and vegetables, but the percentages of those who complied with national nutrition guidelines were low (11). Finally, there is evidence that the BMI of the parent is also associated with the BMI of the children: children with a higher BMI are more likely to have parents with a higher BMI as well (14–17).

Childhood eating habits usually persist into adulthood (2); therefore, it is essential that children develop a healthy eating behavior early in their lives. The World Health Organization (WHO) highlights the need to promote a healthy diet, in particular, promoting fruit, vegetables, and whole grains consumption; and decreasing consumption of excessive sugar and salt and saturated and trans fats (18). Many countries provide dietary guidelines to aid in promoting healthy eating (19–21), including Switzerland (22, 23), where about 20% of children are overweight or obese (24). Like other countries, the Swiss Society for Nutrition (SSN) issued recommendations on what food categories to eat and in what quantities and frequencies one should eat.

Despite efforts to promote healthy eating through the nutrition guidelines, results from the most recent study of nutrition behavior among adults in Switzerland showed that more public health effort is needed to improve eating patterns toward a more healthy behavior. The study showed that while 81% of participants had heard of a food pyramid, only 35% knew of the Swiss food pyramid (24). Regional differences were found, with the French-speaking region being more aware than the Italian- and German-speaking regions (24). A study in the French-speaking part of Switzerland concluded: “the issuing of the Swiss dietary guidelines had little if no impact at all regarding healthy eating,” and suggested that the guidelines should be promoted more effectively (25). Given that parents are the ones who generally determine the food consumption of the children through purchase and preparation of food (26–29), these results are of interest.

Several studies have shown that even when guidelines are known, adherence to them is generally low among adults and adolescents in Switzerland, who are consuming too little vegetables and fruit and overconsuming meat (30–33). Accordingly, a first study (Study 1), measuring adherence to guidelines from a representative sample of children aged 6–12 in Ticino, Switzerland, found low adherence to the SSN guidelines (34). Other studies showed that children did not meet the guidelines for fruit and vegetables and for physical activity (35–37). Data from studies in other countries (i.e., Greece, Italy, Spain, and Turkey) also found that the adherence of the children to nutritional guidelines was low (38, 39). In particular, in northern Italy, which borders Ticino, Switzerland and where the diet is similar to that of our study population, the percentage of children not adhering to the Mediterranean Diet guidelines was over 80% (40).

Given that the behaviors of the children tend to persist in adulthood, and that both children and adults in Switzerland have been found to have low adherence to the guidelines for healthy nutrition, the objectives of the present study (Study 2) were 3-fold. First, the objective was to describe the eating behavior of the Ticino children in terms of adherence to the SSN Guidelines in a second cross-sectional representative sample 2 years after Study 1, noting any changes. Second, to measure the adherence to nutrition guidelines in this second sample and compare the results between the two studies. Third, to assess the associations of factors of the children (gender, age, BMI) and parental factors (gender, BMI, level of education) with adherence of the children to the SNN guidelines.

## Methods

### Study Design and Sample

A cross-sectional study with children ages 5–12 and their parents was conducted in Ticino, Switzerland in 2012. Children and parent dyads who enrolled in a freely available Social Marketing intervention promoting healthy diets were included. The program, in its 2nd edition, was promoted through schools and newspapers to parents and children following the same methods used in the 1st edition of 2010 [see (34, 41)]. A brochure with an enclosed letter for parents providing information on the study and a link to the registration page on the project website was distributed to every child at school. Additional posters were distributed and placed in the corridors and on parent information boards in the schools. Finally, media outlets also published a call for and information about the recruitment. During the registration, parents and children also provided their consent to participate. Children–parent dyads were included if they both consented to participate, provided baseline data (*n* = 331), and the child was between 5 and 12 years of age (*n* = 19 participants aged 13 years were excluded). The intention was to recruit both families that needed to improve their behaviors of the families, as well as families that needed to maintain their already positive behaviors. Based on the findings from Study 1, where on average 23.7% of children adhered to the SSN guidelines (34) and on the findings from previous studies with a similar population, where the adherence to guidelines was 10% (42), 13.1% (30), and 19.6% (40), the power analysis with an expected prevalence of 16.6% was computed (by averaging the means of the four previous studies). By predicting a type I error of 5% and a type II error of 80%, the minimum sample size required was 261 children. The final sample included 312 children (55.0% girls) aged 5–12; mean age (M) = 8.7 years, SD = 1.7.

In this study, the levels of adherence to the SSN guidelines are described and are compared to the levels found in Study 1, which was conducted in 2010 with a sample from the same population (34). In the first study, 568 children were included (50.5% girls) aged 6–12; M = 8.5 years, SD = 1.9. The associations of children- and parent-related factors with adherence of the children to the guidelines and to the consumption of six food categories, namely (1) fruit; (2) vegetables; (3) milk and dairy products; (4) cereal; (5) sweets, snacks, and soft drinks (SSD); and (6) proteins, is also tested.

### Measures

The data of parents were collected through an online survey that included gender, height, weight, and level of education. Children received a paper-based survey and were asked to indicate their gender, date of birth, height, and weight. These variables were included in the analyses as covariates. The surveys used for parents and children were based on validated surveys, such as the PACE study, IPAQ short form, and “*Diamoci una Mossa*,” and on existing surveys used by the Canton of Ticino. The surveys were tested with the target audience, and adjusted for ease of completion, wording, and format (41).

#### BMI

The height and weight of the children were provided by the parents of the children. The recommendation was that they measure the child without clothes and without shoes. The BMI of the children and the parent were calculated as continuous by using the standard formula weight (kg)/[height (m)]^2^. Next, BMI was classified as 0 = Healthy weight, 1 = Underweight, 2 = Overweight, and 3 = Obese. The classification of BMI of the children followed the age- and gender-specific criteria from the U.S. Centers for Disease Control and Prevention, previously validated with Swiss children aged 6–12 (43), and used in the reference study in this population (34).

#### Level of Education

The level of education of the parent was assessed by asking participants which education had the parent completed (elementary, middle, high school; professional school; bachelor, master, or doctoral degrees; or other). The distinction was made between low and high education levels. Participants who had completed at least a bachelor degree were classified into 0 = High level of education, and those who had completed elementary to professional school were categorized into 1 = Low level of education. Participants who replied “others” were assessed individually and classified accordingly.

#### Adherence to Guidelines of the Swiss Society for Nutrition

The frequency of consumption of six food categories of the children (i.e., fruit; vegetables; milk and dairy products; cereal; SSD; and proteins) was collected through a 2-day food record completed by the children. Children completed the 2-day food record on 2 random days, including both weekdays and weekends. The data for this study were collected on 2 days due to the complaints of parents and children regarding the burden of the surveys used in a previous study (41). Hence, for this one, a new instrument collecting data for 2 days, was developed based on the evidence available at that time for other instruments used for dietary assessment (i.e., FFQs, 24 h recall, etc.) (44–48). This instrument was tested in this population and showed that children reliably report their food consumption (49). Since children in Ticino mostly eat at home (including lunch, which is not provided in most schools, and children go home to eat), most meals recorded were consumed at home.

Over 2 days, six times per day, at the main meals (breakfast, lunch, and dinner), and snack moments (morning, afternoon, and after dinner) the children reported what they had eaten by ticking “YES” or “NO” in a list of 11 food groups commonly consumed in this community. Given the notorious difficulties of children (and adults) to accurately report portion sizes (46, 49–53), in this study, the focus was on whether children ate a food group or not, rather than on the amount eaten.

For analysis, every choice was coded as 0 = No and 1 = Yes. A score of daily food consumption was calculated by summing the frequency of consumption of the six meals (from the 2 days) and dividing the result by two (the number of days that the children completed the record). For fruit, vegetables, milk and dairy products, and proteins, the average daily scored varied from 0 to 6 times per day. For cereal and SSD, the mean daily scored ranged from 0 to 12 times per day. This is because children reported on two types of cereal: whole grains (an item) and starchy food (another item), just as the intake of sweets, snacks (an item), and soft drinks (another item) were registered separately too, but combined for this analysis. After the average daily score of food consumption was calculated, adherence to the guidelines (0 = No; 1 = Yes) was computed. Below it is illustrated how the six food groups (outcome-measures) were calculated according to the SSN guidelines.

#### Fruit

A child was categorized as adhering to the SSN guidelines if their consumption was within the range proposed by the SSN for food consumed daily. Children who scored ≥2.0 times/day in fruit consumption were classified as 1 = Yes (adhered to the guidelines). Children who scored <2.0 times/day in fruit consumption were classified as 0 = No (did not adhere to the guidelines).

#### Vegetables

Children who scored ≥3.0 times/day in vegetable consumption were categorized as 1 = Yes and those who scored <3.0 times/day were categorized as 0 = No.

#### Milk and Dairy Products

According to the SSN guidelines, a 6-year old child should eat three portions of dairy daily. Thus, a 6-year old child was classified as adherent to the guidelines if they scored 3.0 times per day, 1 = Yes. Children who scored <3.0 or >3.0 times/day were considered non-adherents, 0 = No. The same reasoning was applied to all children according to their age.

#### Cereal

Children who consumed cereal between 3.0 and 4.0 times/day met the guidelines (1 = Yes) and children who scored <3.0 or >4.0 in cereal consumption did not meet the SSN guidelines (0 = No).

#### SSD

Consumption of SSD <1.0 times/day was considered adherent to the guidelines (1 = Yes) and consumption of SSD ≥1.0 times/day was considered non-adherent (0 = No).

#### Proteins

Information on the consumption of proteins was collected through an item about intake of meat, fish, and eggs of the children (0 = No; 1 = Yes). Thus, it was not possible to measure the consumption of the three types of proteins separately. However, a variable indicating the mean consumption of proteins of the children daily was calculated, which is a proxy of the daily frequency recommended by the SSN. For example, according to the SSN, children aged 6 years should eat proteins 8.0 “times”/week (five portions of meat, one portion of fish, and two eggs), which corresponds to 1.2 times/day. Therefore, children who consumed proteins 1.0 times/day were considered as meeting the guidelines (1 = Yes). Children who consumed >1.0 or <1.0 times/day were considered as non-adherent (0 = No).

### Statistical Analyses

Descriptive statistics were used to portray the characteristics of the analysis sample (see Table 1). In addition, Chi-square tests were performed to assess differences in characteristics between children who did adhere (1 = Yes) or did not adhere (0 = No) to the guidelines of the SSN (see Supplementary Table 1). The associations of children- and parent-related factors with adherence of the children to the SSN guidelines regarding the consumption of six types of foods were assessed by logistic regression analyses, conducted in two steps. First, associations between children-related factors (i.e., gender, age, and BMI) and adherence to the SSN guidelines were assessed through six models (Models 1a−6a) reflecting the six outcomes: (1) fruit, (2) vegetables, (3) milk and dairy products, (4) cereal, (5) SSD, and (6) proteins (see Table 2A). Second, the associations between parent-related factors (i.e., gender, BMI, and level of education) and adherence to the SSN guidelines were assessed, also through six models (Models 1b−6b), reflecting the six outcomes (see Table 2B). All the regression models were simultaneously adjusted for the covariates. The percentage of missing values among children were: age (0.3%), BMI (13.5%), and family structure (21.8%); among parents: gender (23.1%), BMI (23.1%), and level of education (23.1%), and among outcomes: SSD (0.3%), and proteins (0.3%). All analyses were performed with the Statistical Package for Social Sciences (SPSS) version 21.0 for Windows (IBM Corp, Armonk, NY).

**Table 1:** Descriptive characteristics of the analysis sample at baseline (*n* = 312), Switzerland 2012.

**Characteristics**	**Total**		
	**n (%)**	**Mean (SD)**	**Missing n (%)**
**Children**			
Age (Years)		8.7 (1.7)	1 (0.3)
Age categories (Years)			
5–6	55 (17.7)		
7–9	173 (55.6)		
10–12	83 (26.7)		
Gender			0 (0.0)
Male	140 (45.0)		
Female	172 (55.0)		
BMI (Kg/m^2^)		16.8 (2.8)	42 (13.5)
BMI categories			
Underweight	15 (5.6)		
Healthy weight	192 (71.1)		
Overweight	49 (18.1)		
Obese	14 (5.2)		
**Parents**			
Age (Years)		42.1 (4.7)	6 (1.3)
Gender			67 (21.5)
Male	44 (18.0)		
Female	201 (81.0)		
BMI (Kg/m^2^)		23.0 (3.9)	72 (23.0)
BMI categories			
Underweight	7 (2.9)		
Healthy weight	184 (76.7)		
Overweight	36 (15.0)		
Obese	13 (5.4)		
Level of Education			72 (23.1)
Low	161 (70.0)		
High	79 (30.0)		
Nationality			67 (21.5)
Swiss	199 (81.2)		
Non-Swiss	46 (18.8)		
**Children adherence to the Swiss dietary guidelines**			
Fruit			0 (0.0)
Yes	142 (45.5)		
No	170 (55.5)		
Vegetables			0 (0.0)
Yes	14 (4.5)		
No	298 (95.5)		
Milk & Dairy Products			0 (0.0)
Yes	21 (6.7)		
No	291 (93.3)		
Cereal			0 (0.0)
Yes	154 (49.4)		
No	158 (50.6)		
Sweets, Snacks, & Soft Drinks (SSD)			1 (0.3)
Yes	39 (12.5)		
No	272 (87.5)		
Proteins			1 (0.3)
Yes	107 (34.4)		
No	204 (65.6)		

*SD, Standard Deviation; BMI, Body Mass Index*.

**Table 2A:** Logistic regression analyses of the associations between children-related factors and adherence of the children to the guidelines of the Swiss Society of Nutrition (*n* = 287), Switzerland 2012.

	**Model 1a^**a**^**	**Model 2a^**a**^**	**Model 3a^**a**^**	**Model 4a^**a**^**	**Model 5a^**a**^**	**Model 6a^**a**^**
**Children's factors**	**Fruit**	**Vegetables**	**Milk & Dairy Products**	**Cereal**	**SSD**	**Proteins**
	**OR (95% CI)**	**OR (95% CI)**	**OR (95% CI)**	**OR (95% CI)**	**OR (95% CI)**	**OR (95% CI)**
Gender (0 = Male)^b^						
Female	0.84 (0.53–1.35)	0.95 (0.29–3.14)	1.21 (0.52–2.80)	1.04 (0.65–1.67)	0.53 (0.25–1.10)	1.62 (0.97–2.69)
Age (Years)						
4–12	0.97 (0.85–1.11)	1.32 (0.88–1.98)	0.79 (0.63–1.00)	1.00 (0.87–1.31)	0.89 (0.74–1.08)	0.96 (0.83–1.11)
BMI (Kg/m^2^)						
Continuous	1.10 (1.00–1.21)	1.23 (0.89–1.68)	1.00 (0.86–1.17)	1.07 (0.97–1.17)	0.96 (0.85–1.08)	1.15 (1.03–1.28)^*^

a *Each model has been simultaneously adjusted for children's factors: gender, age, and BMI*.

b *References groups are equal to zero. SSD, includes sweets, snacks, & soft drinks; Proteins, includes meat, fish, or eggs*.

* *P < 0.05*.

**Table 2B:** Logistic regression analyses of the associations between parent-related factors and adherence of the children to the guidelines of the Swiss Society of Nutrition (*n* = 287), Switzerland 2012.

	**Model 1b^**a**^**	**Model 2b^**a**^**	**Model 3b^**a**^**	**Model 4b^**a**^**	**Model 5b^**a**^**	**Model 6b^**a**^**
**Parental factors**	**Fruit**	**Vegetables**	**Milk & Dairy Products**	**Cereal**	**SSD**	**Proteins**
	**OR (95% CI)**	**OR (95% CI)**	**OR (95% CI)**	**OR (95% CI)**	**OR (95% CI)**	**OR (95% CI)**
Gender (0 = Male)^b^						
Female	0.68 (0.35–1.36)	5.08 (0.69–37.11)	0.89 (0.27–2.93)	1.22 (0.62–2.40)	1.09 (0.39–3.05)	2.06 (1.00–4.17)
Level of Education (0 = High)						
Low	1.63 (0.95–2.82)	1.12 (0.25–4.96)	1.73 (0.68–4.40)	1.87 (1.08–3.24)^*^	1.90 (0.86–4.19)	0.91 (0.51–1.63)
BMI (Kg/m^2^)						
Continuous	1.08 (1.00–1.17)	1.54 (1.02–2.32)^*^	0.97 (0.87–1.08)	1.01 (0.94–1.08)	1.02 (0.91–1.14)	1.05 (0.97–1.14)

a *Each model has been simultaneously adjusted for parents' factors: gender, level of education, and BMI*.

b *References groups are equal to zero. SSD, includes sweets, snacks, & soft drinks; Proteins, includes meat, fish, or eggs*.

* *P < 0.05*.

### Ethics Statement

The study was reviewed by the Ethics Committee of the Canton Ticino who deemed it exempt in accordance with Swiss law. The recommendations in the Helsinki Declaration were followed. Parents and their children received written explanation about the project and provided an online informed consent. The questionnaires and food records were completed on a voluntary basis, and confidentiality of the responses was guaranteed. Data were anonymized prior to the analyses.

## Results

### Characteristics of the Participants

The analysis sample for the present study included 312 children who completed the 2-day food record at baseline, M = 8.7 years (SD = 1.7). The majority was classified as having a healthy BMI (71.1%). Overweight children corresponded to 18.1%, obese to 5.2%, and 5.6% were underweight (see Table 1). Regarding characteristics of the parent, 81.0% were mothers, M = 42.1 years (SD = 4.7), and 30.0% had a high level of education. The majority was classified as having a healthy BMI (76.7%), 15.0% as overweight, 5.4% as obese, and 2.9% as underweight.

### Adherence With SSN Guidelines and Comparison With the Previous Study

The first objective was to measure adherence to nutrition guidelines in this second sample and compare the results between the two studies. In this study, the consumption of six types of foods of the children did not meet the SSN guidelines. Adherence rates were 4.5% for vegetables, 6.7% for milk and dairy products, 12.5% for SSD, 34.4% for proteins (meat, fish, and/or eggs), 45.5% for fruit, and 49.5% for cereal. For fruit, overconsumption was not distinguished, as it is not considered harmful; all the non-adherents were under-consumers. The majority of children who did not adhere to the guidelines of vegetable consumption (99.6%), milk and dairy products (96.0%), and cereal (76.5%) were under-consumers. For SSD, all non-adherents were over-consumers. For proteins, almost all non-adherents (75.5%) were over-consumers.

**Figure 1:**
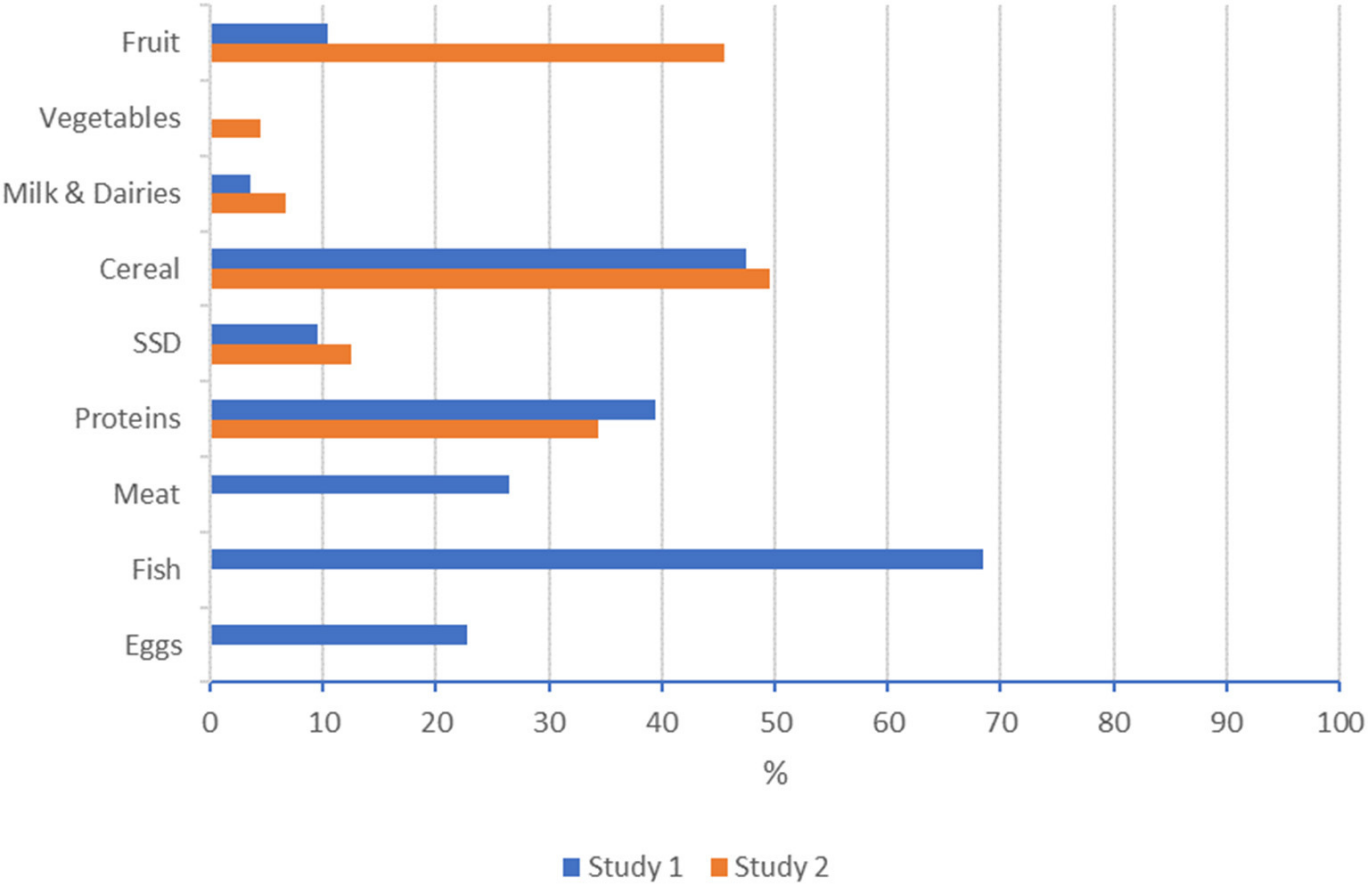
Comparison of adherence to SSN guidelines between Study 1 and Study 2, Switzerland 2012.

As shown in Figure 1, compared to Study 1, an increase in adherence with the recommendations was found in this study (Study 2) for vegetables (0% in Study 1 vs. 4.5% in Study 2); fruit (10.4 vs. 45.5%) milk and dairy products (3.5 vs. 6.7%), SSD (9.5 vs. 12.5%), and cereal (47.9 vs. 49.5%). For proteins, Study 1 showed the following percentages for meat (26.9%), fish (68.5), and eggs (22.7%), while in Study 2, the adherence rate for all three sources of proteins combined was 34.4%, which is slightly lower than the mean of the three sources in the previous study (39.4%).

### Factors Associated With Adherence to SSN Guidelines

The second objective of this study was to assess whether there is an association between individual factors of the children (gender, age, and BMI) and adherence to guidelines. Table 2A shows the results from the logistic regression analysis, which was used to investigate the associations between children-related factors and adherence to the SSN of food consumption (i.e., fruit, vegetables, milk and dairy products, cereal, SSD, and proteins). Compared to Study 1, where increasing age was associated with a decrease in the adherence to the guidelines for cereal, and dairy consumption, and an increase in egg consumption (part of the protein intake), and girls had higher adherence to the guidelines for fruit and meat consumption, in this study, factors of the children were overall not associated with adherence to the guidelines.

However, in both studies, increased BMI of the children was associated with higher adherence to the guidelines for protein consumption (in Study 1, specifically meat consumption). In Study 1 (BMI available in categories), obese children were about twice as likely to adhere to meat consumption as compared to children with a healthy BMI (OR = 1.96; 95% CI = 0.88–4.37; *P* = 0.06). In Study 2 (BMI available continuous), for every 1 Kg/m^2^ increase in BMI of the children, they were 15% more likely to adhere to the SSN guidelines, regarding the protein intake (OR = 1.15; 95% CI = 1.03–1.28; *P* < 0.05).

The third objective of this study was to assess whether there is an association between individual parental factors (gender, BMI, and level of education) and adherence of the children to guidelines. Table 2B shows the logistic regression results of the associations between parent-related factors and adherence to the SSN guidelines. BMI of the parent was significantly associated with adherence of the children to the guidelines regarding vegetable consumption. That is, for each 1 Kg/m^2^ increase in BMI of the parent, children were 54% more likely to adhere to the guidelines (OR = 1.54; 95% CI = 1.02–2.32; *P* < 0.05). Moreover, children whose parents had a low level of education were 87% more likely to adhere to the SSN guidelines for the consumption of cereal compared to children whose parents had a high level of education (OR = 1.87; 95% CI = 1.08–3.24; *P* < 0.05). While in the previous study, the gender of the parent was also a factor that influenced fruit consumption of the children (children that participated with their mothers and not fathers had higher adherence to these guidelines), in this study, no association with this variable was found.

## Discussion

This study shows that adherence of the children to SSN guidelines was overall low and that most children consumed too little fruit, vegetables, cereal, and milk and dairy products, and too much SSD and proteins in comparison with the amounts recommended by the SSN guidelines. Despite some improvement, these findings are consistent with the findings from Study 1, previously conducted among children in Ticino (34). The results are also consistent with other studies with a similar population (10, 36, 38–40). As in the first study, all non-adherers for SSD were over-consumers, as well as the majority of non-adherers for proteins were over-consumers (in the previous study, for meat 72% were over-consumers, while for eggs 71% were under-consumers). On the contrary, in both Study 1 and Study 2, the majority of the children who did not adhere to the recommendation for fruit, vegetables, cereal, and dairy consumption were under-consumers (for fruit, overconsumption was not distinguished, as it is not considered harmful).

Regarding the factors associated with adherence of the children to nutrition guidelines, contrary to Study 1, in this study, it was found that the more overweight the parent, the more children adhered to these guidelines. This may suggest that overweight and obese parents may better understand the risks of these conditions and may want to protect their children. Indeed, other studies have also shown that parental feeding practices and modeling had possibly more influence on the food consumption of the children than parental food consumption itself (13, 54, 55). The influence of parents was also seen in the finding that children whose parents had a low level of education were more likely to adhere to the guidelines for cereal consumption. While a European study showed that overall children whose parents had a lower level of education were less likely to eat vegetables, fruit, pasta, and other low-sugar and low-fat foods (10, 13, 56), according to another study, children whose parents have high education and thus a higher income, have a lower-quality diet, as parents do not have time for food preparation and children often eat outside, with less parental control (57). Further, higher educated parents might think that cereals are not a good source of food, as the idea of pasta causing people to get overweight or obese is quite spread, and parents might thus limit food access with the aim of preventing overweight (58). Also, pasta and other cereals might be less expensive than proteins or vegetables, thus constituting the base of lower-education parent home meals. However, as the economic status of the parents was not analyzed, the authors are not able to provide definite conclusions in this regard. Apart from these two exceptions, overall parental factors were not significantly associated with adherence of the children to guidelines, in line with other research (13). This may suggest that while parents do have a role in what children eat, there are other factors that influence the behavior of the children [such as peer influence, parental modeling, rules in the home about eating (59)].

Further efforts are needed in order to promote healthy eating to children and achieve adherence to nutritional guidelines. Indeed, while there is a small improvement compared to a previous study with a sample of the same population, the results of this study show that children still consume too little fruit, vegetables, cereal, and milk and dairy products, and too much SSD and proteins. In line with other research (25), these results suggest that the SSN guidelines do not have the expected impact on healthy eating and that other measures might be more effective on food purchasing and food eating behaviors (i.e., measures such as changes in prices). Previous studies have shown that, even when known, adherence to the SSN guidelines is low (30–33). Moreover, in another pilot study conducted with the same child population, knowledge of the dietary guidelines at baseline was about 40% (60). In order to increase the likelihood of success, interventions that use more holistic approaches, such as Social Marketing, and that consider and intervene at different levels (individual, environmental, policy) are recommended (61–66). There is a need for behavior change interventions, that is, interventions whose aim is not only to educate but also to achieve actual behavior change. These interventions should be family and population based. Since children in Switzerland eat primarily at home, and since there are already interventions that take place at school, school interventions are not the object of recommendations of the authors. Future studies should also use consistently the same measurement tools, in order for comparisons to be meaningful. At the environmental level, governments should further discuss grocery store food presentations, making healthier food more visible and less expensive.

The strengths of this study include the reliance on a previously tested measurement tool in this population that reflects the food choices of the population. This also allowed to compare data with previous data from the same population, using the same sampling technique. Another strength is that our sample includes children from all BMI categories and not only overweight and obese children. This afforded an understanding of what healthy weight children eat and debunk any misunderstanding that they are more likely to follow nutrition guidelines.

The authors acknowledge several limitations. First, the protein intake could not be divided into meat, fish, eggs, and vegetable proteins, as was the case of the first study, because of the way the item used to measure protein consumption was constructed. Second, a critique, similar to studies using such broad recruitment methods and few exclusion criteria, is that participation in the study was voluntary. This might suggest that participants were more conscious about the health consequences of poor nutrition compared to the rest of the population. If this was the case, it is not a limitation of this study, but rather a call to action, since the results show a low adherence to guidelines even in participants that might be considered more motivated and aware of dietary guidelines than the general population. Third, the authors acknowledge that missing data is frequent in observational studies. The proportions of 13 and 23% missing values in BMI in children and their parents, respectively, might have led to selection bias. Thus, our results should be interpreted with caution. Moreover, the possibility of unmeasured and measured cofounder factors, such as parental income and employment status, could not be ruled out. However, parental level of education was taken into account, which is considered as a good proxy for socioeconomic status and is usually widely accepted (67). Indeed, an Australian longitudinal study showed that parental level of education plays a great role in the food consumption of the children, even when parental work status was taken into account. This study showed that higher parental levels of education had a protective effect on the food consumption of the children, even among unemployed parents, or parents with a part-time job. Further, the tool used to collect data (2-day food record), only recorded 2 days of food consumption of the children. However, based on a study comparing a 7-day food diary with this 2-day food record, data showed that this tool was more reliable in collecting eating behavior data of the children (49, 53). Indeed, children and their parents were asked to independently record food consumption of the children using a 7-day food diary and the 2-day food record used in this study. The diary and food records completed by parents were then compared to those completed by their children, and agreement was measured. While for both the 7-day food diary and the 2-day food records, the results showed a high agreement between parents and children, for the 2-day food record, the agreement was higher than that for the 7-day food diary. A future study could compare the reliability of the 2-day food record over 3–5 days. Finally, portion sizes were not recorded, but the evidence shows that collecting portion sizes is challenging for both adults and children (46, 49–53); thus, new food consumption collecting tools such as this one are urgently needed.

In conclusion, adherence to food guidelines remains low in Ticino, Switzerland. Measures to promote healthy eating are necessary and should incorporate individual, environmental, and policy approaches. Future research should focus on regularly and systematically assessing adherence of the children to nutritional guidelines, possibly at the national level and using the same eating behavior measurement tool, providing evidence of needs for behavior change interventions and health policy.

## Data Availability Statement

The raw data supporting the conclusions of this article will be made available by the authors upon request, without undue reservation.

## Ethics Statement

The study was reviewed by the ethics committee of the Canton Ticino who deemed it exempt it in accordance with Swiss law. The recommendations in the Helsinki Declaration were followed. Parents and their children received written explanation about the project and provided an online informed consent.

## Author Contributions

NR and LSS formulated the research question and designed the study. NR carried out the study and collected the data. RNAS analyzed the data. All authors wrote the article and approved the final manuscript.

## Conflict of Interest

The authors declare that the research was conducted in the absence of any commercial or financial relationships that could be construed as a potential conflict of interest.
