# Supplementary material for: Policy and Food Consumption: What Nutrition Guidelines Are Swiss Children Meeting and What Determines Adherence?
Source: Front Nutr. 2021 Jun 4;8:641799. doi: 10.3389/fnut.2021.641799 (PMC8211762; doi:10.3389/fnut.2021.641799)
Supplement: Supplementary file 1 [file Table_1.docx]

**Supplementary Table 1** Bivariate analysis of the factors associated with children’s adherence to the guidelines (yes; no) of the Swiss Society of Nutrition (n=331), Switzerland 2012

|  |  | Fruit | | Vegetables | | Milk & Dairy Products | | Cereal | | SSD | | Proteins | |
| --- | --- | --- | --- | --- | --- | --- | --- | --- | --- | --- | --- | --- | --- |
|  | Total | Yes | P ^a^ | Yes | P ^a^ | Yes | P ^a^ | Yes | P ^a^ | Yes | P ^a^ | Yes | P ^a^ |
|  | n | n (%) |  | n (%) |  | n (%) |  | n (%) |  | n (%) |  | n (%) |  |
| *Children* |  | |  |  |  |  |  |  |  |  |  |  |  |
| Age categories (Years) |  | | .631 |  | .238 |  | .541 |  | .952 |  | .281 |  | .622 |
| 5–6 | 55 | 22 (40.0) |  | 3 (5.5) |  | 2 (3.6) |  | 28 (51.0) |  | 5 (9.0) |  | 19 (34.5) |  |
| 7–9 | 173 | 82 (47.4) |  | 10 (5.8) |  | 12 (6.9) |  | 85 (49.0) |  | 19 (11.0) |  | 56 (32.4) |  |
| 10–12 | 83 | 38 (45.8) |  | 1 (1.2) |  | 7 (8.4) |  | 40 (48.2) |  | 14 (17.0) |  | 32 (38.6) |  |
| Gender |  |  | .695 |  | .481 |  | .793 |  | .666 |  | .056 |  | .060 |
| Male | 140 | 62 (44.3) |  | 5 (3.6) |  | 10 (7.1) |  | 71 (50.7) |  | 12 (8.6) |  | 56 (40.0) |  |
| Female | 172 | 80 (46.5) |  | 9 (5.2) |  | 11 (6.4) |  | 83 (48.3) |  | 27 (15.8) |  | 51 (29.9) |  |
| BMI categories |  |  | .718 |  | .247 |  | .560 |  | .356 |  | .558 |  | .012 |
| Underweight | 15 | 8 (53.3) |  | 2 (13.3) |  | 1 (6.7) |  | 9 (60.0) |  | 1 (6.7) |  | 11 (73.3) |  |
| Healthy weight | 192 | 92 (47.9) |  | 9 (4.7) |  | 12 (6.3) |  | 94 (49.0) |  | 23 (12.0) |  | 61 (32.0) |  |
| Overweight | 49 | 21 (42.9) |  | 1 (2.0) |  | 6 (12.2) |  | 22 (45.0) |  | 8 (16.3) |  | 16 (32.7) |  |
| Obese | 14 | 5 (35.7) |  | 0 (0.0) |  | 1 (7.1) |  | 4 (28.6) |  | 3 (21.4) |  | 4 (28.6) |  |
| *Parents* |  |  |  |  |  |  |  |  |  |  |  |  |  |
| Gender |  |  | .326 |  | .598 |  | .882 |  | .881 |  | .756 |  | .132 |
| Male | 44 | 17 (38.6) |  | 2 (4.5) |  | 3 (6.8) |  | 22 (50.0) |  | 6 (13.6) |  | 19 (43.2) |  |
| Female | 201 | 94 (46.8) |  | 6 (3.0) |  | 15 (7.5) |  | 98 (48.8) |  | 24 (12.0) |  | 63 (31.3) |  |
| BMI categories |  |  | .350 |  | .472 |  | .246 |  | .945 |  | .949 |  | .353 |
| Underweight | 7 | 5 (71.4) |  | 0 (0.0) |  | 0 (0.0) |  | 3 (43.0) |  | 1 (14.3) |  | 3 (43.0) |  |
| Healthy weight | 184 | 85 (46.2) |  | 8 (4.3) |  | 12 (6.5) |  | 89 (48.4) |  | 23 (12.5) |  | 61 (33.2) |  |
| Overweight | 36 | 15 (41.7) |  | 0 (0.0) |  | 5 (13.9) |  | 19 (52.8) |  | 5 (13.9) |  | 15 (41.7) |  |
| Obese | 13 | 4 (30.8) |  | 0 (0.0) |  | 1 (6.7) |  | 6 (46.2) |  | 1 (7.7) |  | 2 (15.4) |  |
| Level of Education |  |  | .061 |  | .779 |  | .279 |  | .061 |  | .041 |  | .771 |
| Low | 161 | 65 (40.4) |  | 5 (3.1) |  | 10 (8.1) |  | 73 (45.3) |  | 14 (8.7) |  | 52 (32.3) |  |
| High | 79 | 42 (53.2) |  | 3 (3.8) |  | 10 (6.2) |  | 46 (58.2) |  | 14 (17.7) |  | 27 (34.2) |  |
| Nationality |  |  | .478 |  | .644 |  | .812 |  | .408 |  | .495 |  | .834 |
| Swiss | 199 | 88 (44.2) |  | 7 (3.5) |  | 15 (7.5) |  | 100 (50.3) |  | 23 (11.6) |  | 66 (33.2) |  |
| Non-Swiss | 46 | 23 (50.0) |  | 1 (2.2) |  | 3 (6.5) |  | 20 (43.5) |  | 7 (15.2) |  | 16 (34.8) |  |

SD=Standard Deviation. BMI=Body Mass Index. SSD=sweets, snacks, & soft drinks.

^a^ Significance level of differences between children who adhered (yes) or did not adhere (no) to the guidelines of the Swiss Society of Nutrition by Chi-square tests. Bold print indicates statistical significance.
